# Quantitative and Comparative Analysis of Effectivity and Robustness for Enhanced and Optimized Non-Local Mean Filter Combining Pixel and Patch Information on MR Images of Musculoskeletal System

**DOI:** 10.3390/s21124161

**Published:** 2021-06-17

**Authors:** Jan Kubicek, Michal Strycek, Martin Cerny, Marek Penhaker, Ondrej Prokop, Dominik Vilimek

**Affiliations:** 1 Department of Cybernetics and Biomedical Engineering, VSB–Technical University of Ostrava, 17. listopadu 15, 70800 Ostrava Poruba, Czech Republic; michal.strycek@vsb.cz (M.S.); martin.cerny@vsb.cz (M.C.); marek.penhaker@vsb.cz (M.P.); dominik.vilimek@vsb.cz (D.V.); 2 MEDIN, a.s., Vlachovicka 619, 59231 Nove Mesto na Morave, Czech Republic; ondrej.prokop@medin.cz

**Keywords:** musculoskeletal system, image denoising, non-local means, filter robustness, local-means, parameters optimization, segmentation performance

## Abstract

In the area of musculoskeletal MR images analysis, the image denoising plays an important role in enhancing the spatial image area for further processing. Recent studies have shown that non-local means (NLM) methods appear to be more effective and robust when compared with conventional local statistical filters, including median or average filters, when Rician noise is presented. A significant limitation of NLM is the fact that thy have the tendency to suppress tiny objects, which may represent clinically important information. For this reason, we provide an extensive quantitative and objective analysis of a novel NLM algorithm, taking advantage of pixel and patch similarity information with the optimization procedure for optimal filter parameters selection to demonstrate a higher robustness and effectivity, when comparing with NLM and conventional local means methods, including average and median filters. We provide extensive testing on variable noise generators with dynamical noise intensity to objectively demonstrate the robustness of the method in a noisy environment, which simulates relevant, variable and real conditions. This work also objectively evaluates the potential and benefits of the application of NLM filters in contrast to conventional local-mean filters. The final part of the analysis is focused on the segmentation performance when an NLM filter is applied. This analysis demonstrates a better performance of tissue identification with the application of smoothing procedure under worsening image conditions.

## 1. Introduction

The musculoskeletal system comprises a set of organs that allows a person to move (Latin locomotion—hence the name locomotor system). In principle, we can divide this system into a system of muscles, which are their own executors of motion, and a bone (support) system, where, in addition to bones, we can also include joints, ligaments and tendons [1,2,3,4]. In addition to the basic motor functions, this complex system performs many other indispensable tasks, such as upright posture, protection of vital organs, especially the central nervous system and organs in the abdominal cavity, heat generation needed to maintain a constant body temperature, metabolic function protein supply and, finally, communication functions (e.g., the contraction of mimic muscles expresses our feelings, gesticulation is an important part of interpersonal communication) [5,6]. Based on these facts, the musculoskeletal system is substantially important for a range of human activities. Therefore, a proper investigation of these tissues is crucially important for diagnostic information [7,8,9,10].

The main important aspect of the prevention and treatment of potential musculoskeletal disorders is a proper investigation by using imaging systems. Clinically, ultrasound and MR (magnetic resonance) examination are conventionally used [11,12,13]. Magnetic resonance imaging is a non-invasive examination method that has become an irreplaceable part of the complex of imaging methods used by modern medical science. MR is principally based on the changing magnetic moments of atomic nuclei. The patient is placed into a very strong magnetic field, where a short radio frequency pulse is sent and after at its end a magnetic signal is sensed [14,15]. It forms the nuclei of hydrogen atoms in the patient’s body. The signal is then measured and used to reconstruct the image. However, an absolute contraindication is a pacemaker, electronically controlled implants, vascular clamps made of ferromagnetic or unknown material and metal foreign bodies in the eye. Relative contraindications include metal alien bodies, claustrophobia, first trimester of pregnancy, total endoprosthesis (TEP), stents, and clamps up to 6 weeks after implantation [16,17].

MR examination is indicated for the evaluation of muscle and tendon trauma, to distinguish cysts, ganglia, and hematomas, to diagnose hidden fractures, acute chronic osteomyelitis and to assess traumatic and non-traumatic joint changes [18]. Moreover, MR is an excellent method for evaluating bone marrow disease changes; for example, in bone circulatory disorders, in the early stages of fatigue fractures, in inflammation and some malignancies. The most used investigative technique is to determine the T1 (longitudinal) and T2 (transverse) relaxation times, where T1 time is the time constant which determines the rate at which excited protons return to equilibrium, and T2 time represents the time constant which determines the rate at which excited protons reach equilibrium or go out of phase with each other [19]. The basic investigative procedures include a spin–echo sequence, which is a basic examination. Individual tissues have different T1 and T2 times and thus differences in signal strength, which is reflected in the difference in grayscale. Darker structures are hyposignal and the lighter structures are hypersignal, and the images thus obtained are called T1- and T2-weighted images [20,21].

As we reported earlier, magnetic resonance plays an essential role in the musculoskeletal system investigation. In order to perform a proper investigation of the individual issues in this system, the quality of medical images represents a crucial factor. When the image data are corrupted with image noise or artefacts, the extraction of clinically important parameters is limited, and interpretation of the diagnostic information may be misleading. Therefore, image smoothing represents a very important aspect of the algorithms, which are aimed at the extraction of tissues, and its features of interest. In this context, the concept of non-local means (NLM) methods appears to be an effective alternative for image smoothing, when compared with conventional smoothing methods such as average or median image filters, taking advantage of only a local neighborhood of a representative pixel. The main aim of this study is the investigation of a novel NLM approach, taking advantage of pixel and patch similarity information to improve the filtration effect. We mainly study the effectivity of the proposed method for various settings in the contrast with a standard NLM algorithm, as well as conventional local means techniques. We provide the analysis of the filter effectivity and robustness under the influence of selected image noise generators (Gaussian, Rician and Salt and Pepper) with dynamic noise intensity to objectively report the dynamical noise intensity influence on the filtration quality. To justify the noise effect on the quality of musculoskeletal features extraction, we also provide an analysis of the robustness of selected issues of interest identification, when using the proposed filter under various noise effect.

The rest of the paper is organized into the following sections. Section 2 deals with the recent research in the area of local and non-local filtration techniques for image smoothing. Section 3 is focused on the design of the NLM filter with the pixel and patch similarity information. Section 4 is focused on the analysis and results of the NLM filter for dynamical noise influence, statistical analysis of intensity differences and the analysis of the filter application for regional segmentation performance.

## 2. Recent Work

In this section, we are focused on the recent approaches and advances for medical image smoothing. This area belongs to the procedures of image preprocessing, with the focus on enhancing the quality of the image features, which are crucial for a proper diagnosis of the areas of interest of musculoskeletal images. In this context, as a standard, we require the performance of the spatial image area smoothing to reduce a level of image noise and artefacts and, at the same time, we strive to preserve the image edges, which represent substantial information for the identification of tissues.

There are many smoothing methods that have been proposed for the task of edge-preserving image smoothing. Such methods may be classified into two groups. The first group refers to the methods, utilizing local information in the surrounding of a representative pixel, formed by a local window [22,23,24]. In this category, we can mention bilateral filters, average, median, weighted median filters (WMF), anisotropic diffusion (AD) and edge-avoiding wavelet (EAW) [25,26]. One of the substantial limitations of such local filters is producing artefacts in the form of halos along the image edges [27]. This limitation is caused by computing only local statistics for the purpose of filtering. Therefore, it is impossible to control the statistical features of the filtered images [28,29,30,31].

The second group of the filters is global optimization approaches. In this case, the resulting smoothed image is determined as the result of solving a global objective function. Such a function usually involves a data term. A data term procedure does constrain the distance between the original and smoothed image. A smoothness is performed by using a regularization term. In this section, we can mention weighted least square smoothing (WLS) [32,33], fast global smoother (FGS) [34], SD filter [35], and improved dictionary learning with global structure and local similarity preservations [36]. These methods typically overcome some limitations, which are connected with the local filters, such as halo effect and gradient reversals. On the other hand, we should mention that these filters are usually more time consuming when compared with the local based filters [37,38,39,40,41].

Each filter is considered in several ways. Firstly, the effectivity of a filter is very important in the context of removing unwanted image noise and artefacts [42,43]. However, as we mentioned earlier, an important aspect is also computing time, which determines the time efficiency. This factor gains particular importance when processing a batch of images, when time demanding procedures cause significant limitations in such procedures [44,45]. Besides these facts, the filter robustness represents a very important factor, reporting the filter behavior in dynamical image environments caused by dynamic image noise. These characteristics objectively report the filter stability (robustness) in different image conditions, which is one of the main issues of this paper.

In medical image preprocessing, the NLM filter and its variants are frequently used for image denoising and smoothing. The basic idea of NLM is based on the estimation of the mean values of all pixels in the image, which are weighted by similarity of these pixels to the target pixels. This is the major difference when comparing with conventional local mean approaches [46,47]. There are similar modifications of the NLM filter, taking advantage of weighted functions to improve the denoising effect, such as an optimized self-similar patch-based filter [48], an NLM filter with adaptive similarity functions [49], an NLM filter based on recursive calculation of similarity weights [50], and an NLM filter with patch similarity [51]. An interesting study [52] deals with the Rician noise removal by the application of NLM filtering for low signal to noise ratio images.

## 3. Materials and Methods

In this section, we introduce the proposed concept of NLM filter, utilizing pixel and patch similarity information in the application of musculoskeletal MR images. The basic concept of a non-local means algorithm, unlike conventional local means filters, which consider the mean value of the pixel’s neighborhood of a representative pixel, takes a mean value of all the pixels in the image area, weighted by the term, representing the pixel’s similarity to the representative pixel. This approach has been frequently proven to be more effective in terms of detail loss reduction, when compared with the conventional local means approaches.

The model of an ideal noisy image (*Y*) can be represented by the image intensity y(i), which is composed of Gaussian noise n(i). $\left(\sigma^{2}\right)$ stands for Gaussian noise with zero mean value x(i) is the variance and intensity of the image, and (X) is not containing noise. Such an ideal form of the image noise model can be formulated in the following way:

(1) y(i)=n(i)+x(i),x(i)∈X, y(i)∈Y

### 3.1. Original RNLM Algorithm

The principle of the NLM filter is focused on the estimation of $\hat{x}\left(i\right)$ by calculating the weighted average intensity of the considered pixels located in a search window (w(i)) being centered at a pixel (*i*). The estimation of $\hat{x}\left(i\right)$ can be formulated in the following way:

(2) $$\hat{x}\left(i\right)=\underset{j\in Win\left(i\right)}{\sum }w\left(i,j\right).y\left(j\right)$$

In this formulation, $w\left(i,j\right)=\varphi \left(i,j\right)/\left(\underset{\forall j\in Win\left(i\right)}{\sum }\varphi \left(i,j\right)\right)$ stands for normalized weight function, which is given by the distance calculated between the noisy patches located at the pixel (N(Y(i))) and the second pixel (N(Y(j))), belonging to the noisy image (Y). In the next step, the weight function before normalization between pixels *i* and *j* (φ((i,j)) is formulated in the following way:

(3) $$\varphi \left(i,j\right)=exp\left(-\|N\left(Y(i\right))-N\left(Y(j)\right){\|}_{2,a}^{2}\right)/h^{2},\forall j\neq i$$

In this formulation, the term $\|.{\|}_{2,a}^{2}$ stands for the Gaussian weighted Euclidean distance, Y(i), Y(j) stands for the intensity vectors of a local neighborhood of the representative pixels *i* and *j*, *a* is the standard deviation of Gaussian function, and *h* stands for the decay rate of weights. The parameter *h* is substantially important in terms of controlling the degree of smoothing. The image (*I*) is divided into a finite set of nonoverlapping patches, having the same dimension *N × N* pixels. The patch (*Y*) of the neighborhood *3 × 3* between pixels *i* and *j* is defined in the following way:

(4) $$Y\left(i,j\right)=\left[\begin{array}{ccc} I\left(i-2,j-2\right) & I\left(i-2,j\right) & I\left(i-2,j+2\right)\\ I\left(i,j-2\right) & I\left(i,j\right) & I\left(i,j-2\right)\\ I\left(i+2,j-2\right) & I\left(i+2,j\right) & I\left(i+2,j+2\right) \end{array}\right]$$

One of the possible limitations of this approach is the over-weighting of pixel *i*. To avoid this unfavorable effect, the weight (φ((i,j)) is assigned the maximal weight of non-central pixels from the search window. This operation is formulated by this way:

(5) φ(i,j)=max{φ(i,j)},∀j≠i

The original concept of NLM supposes zero Gaussian noise. In order to generalize this concept for MR images, this concept of an NLM algorithm should be adapted to non-zero bias, which is typical case of Rician noise. Thus, the original definition of an RNLM filter [52] is given in the following way:

(6) $$RNLM\left(\hat{x}\left(i\right)\right)=\sqrt{max\left(\left(\underset{j\in Win\left(i\right)}{\sum }w\left(i,j\right).y{\left(j\right)}^{2}\right)-2\sigma^{2},0\right)}$$

In this formulation, $\sigma^{2}$ stands for the variance of Gaussian noise, which can be estimated from the background as: $\sigma =\sqrt{\mu /2}$, where μ denotes the mean value of squared magnitude of the MR image background. One of the significant limitations of an RNLM algorithm is reducing small regions, manifesting as particles, which may represent an important information, such is for instance tiny bone lesions. Therefore, we deal with a novel patch and pixel similarity approach.

### 3.2. RNLM Algorithm with Patch and Similarity Information

The aim of the proposed method is retaining small high-contrast particle details in MR images. This task should be performed by using weighting method, combining patch and pixel similarity information, which is described in this section. In contrast with the original RNLM algorithm [52], it takes advantage of the maximum weight of non-central pixels in the form of self-weight. In this way, it has the same problem as the NLM filter with particle loss.

In the case of presence of such small intensity clusters (particles) in the MR image, their intensity spectrum is significantly different from the image background in a search window. We suppose that the mentioned intensity spectrum difference is significantly higher than the spectrum caused by the image noise. This fact can be taken advantage of in order to mitigate the so-called particle-blurring issue of the NLM filter. Particle-blurring is a typical effect of NLM and RNLM filters, which have a tendency to suppress small particles (spots) in MR images. Since such spots may represent significantly important locations such as cartilage lesions or the early signs of cartilage deterioration, this blurring effect should be compensated. For this reason, the proposed filter utilizes the patch and pixel’s intensity similarity ($\;\varphi {\left(i,j\right)}^{*}$). The proposed method calculates the weight function $\left(\varphi {\left(i,j\right)}^{*}\right)$ as a combination of the patch and pixel similarity level in the following way:

(7) $$\varphi {\left(i,j\right)}^{*}=\varphi \left(i,j\right).\rho \left(i,j\right),i\neq j$$

(8) $$\rho \left(i,j\right)=\frac{1}{1+{\left(\left|y\left(i\right)-y\left(j\right)\right|/Deg^{c}\right)}^{\omega }}$$

In this formulation, φ(i,j) stands for the similarity between patches given by the pixels *i* and *j* and ρ(i,j) stands for the pixel’s function of similarity, formulated as a decreasing function for the intensity spectrum difference |y(i)−y(j)|. This decreasing function ensures the assignment of higher weights for the pixels with intensity closely related to the central pixel. The parameters $Deg^{c}$ and ω ensure control of the position and the slope of transition, respectively. The parameter ρ(i,j) is limited in the range [0;1], which classifies the situation (ρ(i,j)=0) when the intensity pixel (*j*) is significantly different from the central pixel (*i*). Based on these formulations in Equations (6) and (7), only the pixels simultaneously having higher patches and higher levels of similarity are classified as higher weights in the filtering procedure. The self-weight function is defined by the formulation:

(9) $$\varphi {\left(i,j\right)}^{*}=\varphi {\left(i,k\right)}^{*}\vartheta \left(i,k\right)$$

where $k=arg\left\{max_{j}\left\{\varphi {\left(i,k\right)}^{*},\forall j\neq k\right\}\right\}$

(10) $$\vartheta \left(i,k\right)=\left(1+\frac{{\left(2rad_{p}+1\right)}^{2}}{1+{\left(Deg^{c}/\left|y\left(i\right)-y\left(k\right)\right|\right)}^{\omega }}\right)$$

In this formulation, (*k)* stands for the non-central pixel index, having the highest level of similarity to the central pixel (*i*) within a search window, and $\varphi {\left(i,j\right)}^{*}$ represents the maximal weight. When compared with the Equation (7), the function, representing pixel’s similarity ϑ(i,k) is given as an increasing function of the pixel’s intensity difference. Each search window is represented by its radius parameter ($rad_{p}$). The scale factor ϑ(i,k) increases with the absolute pixel’s intensity difference |y(i)−y(k)|. In the case of tiny intensity particles, having a high contrast, where the central pixel (*i*) has a significantly different intensity spectrum than the pixel (k), a higher weight function (ϑ(i,k)≫1) will be assigned. That is the case when |y(i)−y(k)| is higher than $Deg^{c}$. By using this principle, such small clusters of pixels are preserved by the filtering procedure. In the case of the small contrast spots, when the intensity of the central pixel *I* is significantly different from the selected pixel (*k*), higher weights (ϑ(i,k)) should be classified. This situation predetermines the fact that such particles will be classified and thus preserved. The final form of the filter is formulated (by using Equation (6)) in the following way:

(11) $$RNLM^{*}\left(\hat{x}\left(i\right)\right)=\sqrt{max\left(\left(\underset{j\in Win\left(i\right)}{\sum }w{\left(i,j\right)}^{*}.y{\left(j\right)}^{2}\right)-2\sigma^{2},0\right)}$$

In this formulation, the parameter $w{\left(i,j\right)}^{*}$ represents the weight function after the normalization procedure $\varphi {\left(i,j\right)}^{*}$.

## 4. Results

**Figure 1:**
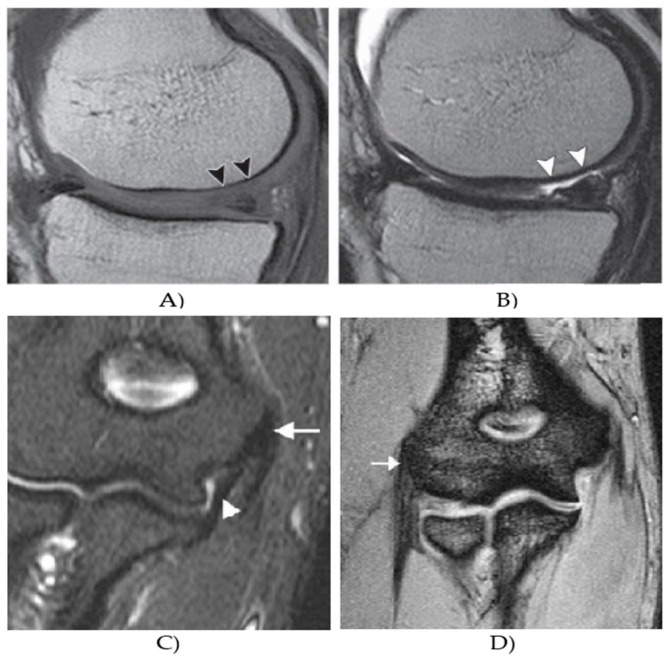
Example from MR datasets: (**A**) T1 weighted image, showing a weak contrast between the cartilage surface and synovial fluid, (**B**) proton density-weighted image, (**C**) coronal fat-saturated proton density-weighted image of elbow muscle, showing a low signal intensity of the common flexor tendon, which is located at the medial epicondyle (arrow), and (**D**) coronal gradient echo image of elbow muscle, showing a normal manifestation of a normal extensor tendon at the lateral epicondyle (arrow).

In this section, we provide a quantitative evaluation and performance analysis of the NLM filter, incorporating the patch and similarity information in the contrast of standard NLM filter and conventional local mean approaches, including average and median filter to demonstrate its robustness in various environment, caused by the effect of additive noise generators with dynamic noise intensity. Since the filtration procedure, providing smoothing of image area is a common part of the object identification from MR images, we also provide a quantitative analysis of a regional segmentation performance, when using the proposed filter. Figure 1 represents an example of the MR database, which we use for the testing.

### 4.1. Musculoskeletal MR Images

For the purpose of analysis, we used three retrospective MR datasets, including the MR cartilage data of fat saturation techniques, proton density-weighted imaging, and shoulder joints images. The datasets used for the filter’s testing are from the public database, The Osteoarthritis Initiative (OAI).

Commonly, the fat saturation technique is used for the MR cartilage imaging. This technique involves the excitation and dephasing of the spinning protons in fat by applying lipid-specific radiofrequency pulse, which is utilized before each repetition of 2D or 3D SE or GRE imaging sequence. A great advantage is increase in the contrast between lipid and non-lipid surfaces, in addition to suppression of the chemical shift artefact. In this study, we have a total of 80 of the cartilage images from the fat saturation technique.

With the proton density-weighted imaging, we can depict the surface of cartilage effects as well as the internal cartilage abnormalities composition. The proton density-weighted imaging techniques provide a reliable investigation of the cartilage morphological assessment as well as menisci and ligaments (ligamentous structures). In our study, we use retrospective data, including 70 MR images of proton density-weighted imaging.

The last dataset comprises a normal anatomy of the elbow muscle individual compartments. We analyze coronal fat-saturated proton density-weighted images. These images are a good demonstration of a common low signal intensity of the common flexor tendon, located at the medial epicondyle. Additionally, we have coronal gradient echo images, which are focused on common extensor tendons at the lateral epicondyle. We have a total of 40 MR images of the elbow, which we use for this analysis.

Table 1 summarizes the acquisition parameters for individual datasets, including FOV (field-of-view), matrix spatial resolution, acquisition time, slice thickness, interslice gap, and scan mode.

### 4.2. Additive Noise Generators

**Figure 2:**
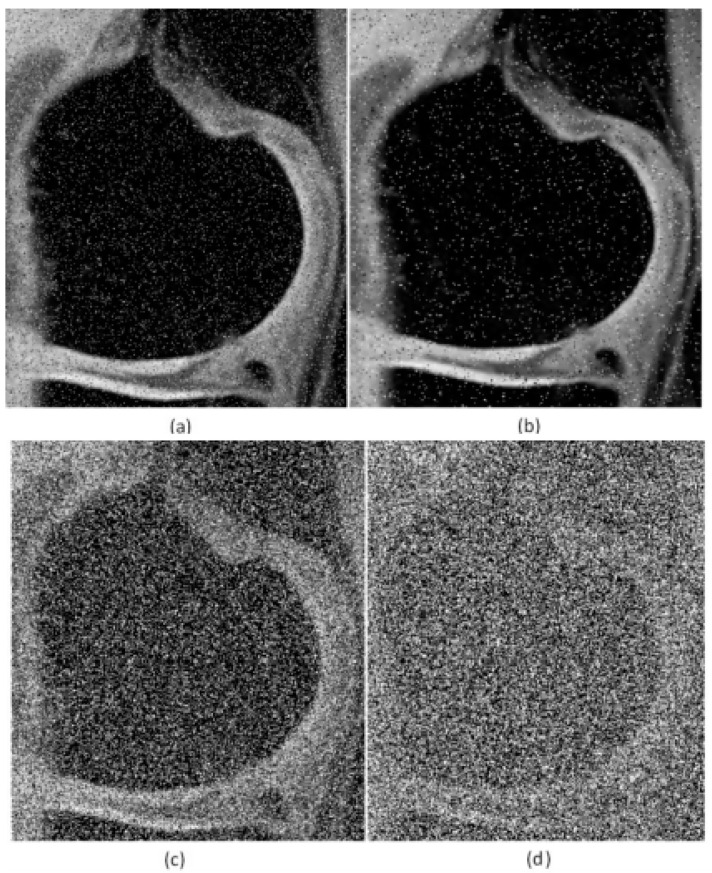
Selected area of interest (RoI) for MR image of articular cartilage with dynamic Salt and Pepper noise intensity (d): (**a**) d = 0.08, (**b**) d = 0.12, (**c**) d = 0.5, and (**d**) d = 0.85.

**Figure 3:**
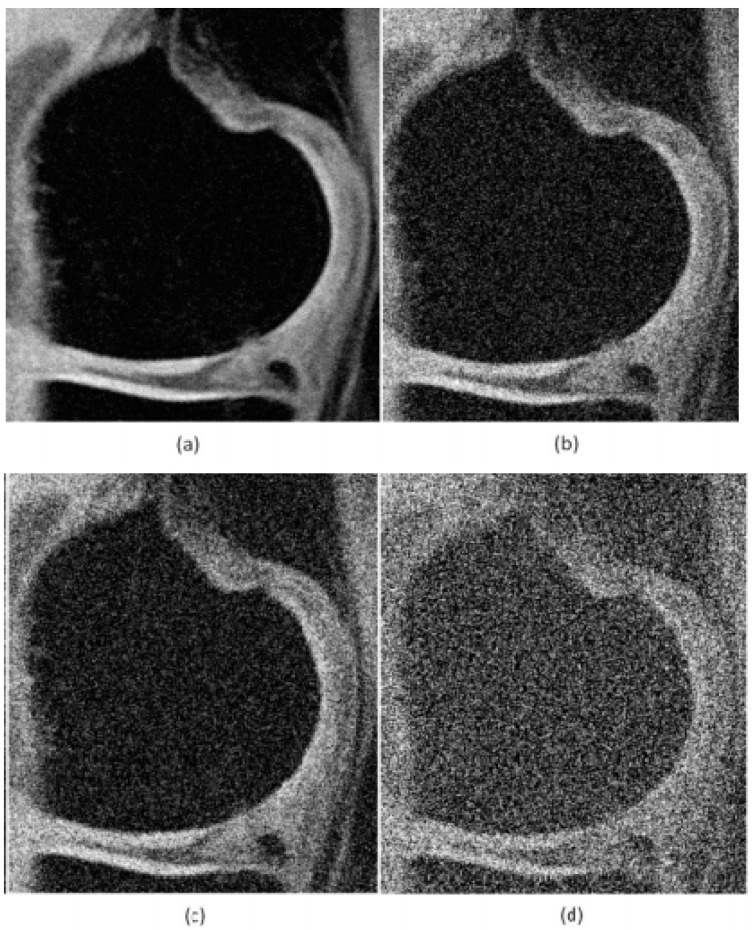
Selected area of interest (RoI) for MR image of articular cartilage with dynamic Rician noise intensity: (**a**) σ=0.02, (**b**) σ=0.09, (**c**) σ=0.15, and (**d**) σ=0.75.

The characteristic performance demonstration of the NLM filter is based on the additive noise generators, including Rician noise, which is typical for the MR images, the impulse noise of type Salt and Pepper, and Speckle noise. All the types of the noise generators are controlled by using their steering parameters, enabling control the noise intensity. The noise generators are implemented in the form of gradual dynamical noise. That allows us to investigate the dynamical features of the smoothing effectivity in the form of robustness characteristics, which provides the information about the filter response in various image conditions. To demonstrate the influence of various forms of noise, we provide examples of MR images of articular cartilage corrupted via Salt and Pepper noise with different noise intensity (Figure 2) and Rician noise (Figure 3).

Rician noise is the most typical model of the image noise, which appears in the real MR images. This noise is derived from Gaussian noise. The signal magnitude can be expressed in the following way:

(12) $$M=\sqrt{{\left(A+n_{1}\right)}^{2}+n_{2}^{2}}$$

In this formulation, *M* stands for the signal magnitude, *A* is the original noise-free image, and $n_{1}$ and $n_{2}$ represent not correlated variables of the Gaussian noise with zero mean value and the same dispersion $\sigma_{n}^{2}$. The probability density function (PDF) for such image is indicated as Rician distribution in the following way:

(13) $$p(M|A,\sigma_{n}^{2})=\frac{M}{\sigma_{n}^{2}}\exp\left(-\frac{M^{2}+A^{2}}{2\sigma_{n}^{2}}\right)I_{0}\left(\frac{AM}{\sigma_{n}^{2}}\right)u\left(M\right)$$

In this formulation, $I_{0}\left(.\right)$ stands for 0th-order modified Bessel function of the first kind and the parameter u(.) represents Heviside step function [53].

Speckle noise is manifested as a granulated texture and causes the gray level average increment in the target area. It is perceived as an unwanted feature. This noise intensity is given by its dispersion (v). This noise can be interpreted by the formulation:

(14) J=I+n*I

In this formulation, *I* stands for the input image, *J* represents the noise distribution in the image, and *n* represents unified zero mean value of the noise in image.

The last considered noise in this study is an impulse noise—Salt and Pepper. This noise is represented by white and black pixels of defined density (d). The noise manifestation predetermines its binary intensity spectrum.

### 4.3. Set up of the NLM Filter and Parameters Optimization

**Figure 4:**
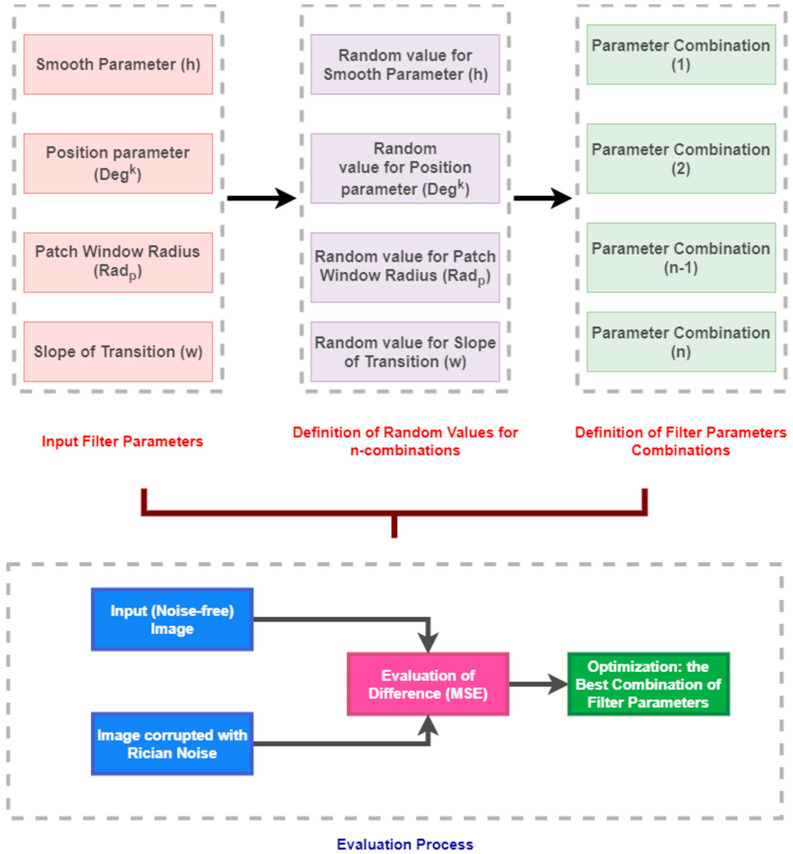
A proposed scheme for the filter parameters optimization bases on the random selection and MSE evaluation.

Herein, we analyze the settings of the proposed filter for MR image smoothing. An important issue of this implementation is a set of the filter parameters, not having deterministically given values. These values can be theoretically set empirically, but more precise way, which we use is focus on an optimization procedure, which will predict and recommend a proper value setting. We use an optimization procedure to find the best combination of the filter parameter bases on the difference evaluation (MSE) cost function (Figure 4).

This filter uses the three following parameters. Their values should be optimized to find the best combination, which correspond with the most effective results of filtration. Firstly, we search for optimal values of parameter (h = $\sigma^{2}\in \langle 0;1\rangle$), which represents the smooth controlling parameter; the next parameter, ($rad_{p}\in \mathbb{Z}$) represents a radius of patch window, $deg^{k}$ controls the position, (k) determines a level of the steepness (deg∈〈0;1〉 and k∈ℤ); parameter ω represents the slope of transition (ω∈ℤ).

To find optimal values for individual parameters, we implemented a set of generators of random values from the defined interval parameters described above. The optimization procedure randomly generates n combinations of the parameters, with the values from defined ranges. For each combination, we evaluated the filter settings effectivity based on the Mean Squared Error (MSE) between the result of the proposed filter and the original noisy-free image. We performed this procedure for 80 records of MR data (20 images from each dataset) corrupted with Rician noise with the settings: σ=[0.05, 0.1, 0.15, 0.2, 0.3]. Finally, for each filter settings, all the values were averaged. Based on the MSE evaluation, we selected the combination of the filter parameters, minimizing MSE function and, thus, difference between original image and the filter output.

**Figure 5:**
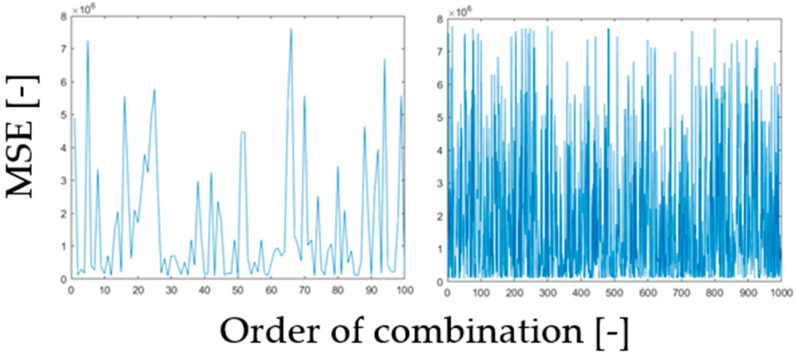
A comparison of MSE evaluation for number of filter parameter’s combinations (n = 100) left and (n = 1000) right for 80 MR records (averaged values for each n).

We evaluated the MSE for each combination (n) of filter parameters. Based on this evaluation, we report the spectrum of MSE values, showing a distribution of the error function for individual parameter’s settings (Figure 5).

We evaluated these characteristics by the minimal value of MSE (Table 2). These results indicate the filter design combination as having the smallest difference between the filter output and native (noise-free). Based on the MSE comparison for individual combination of the filter’s parameters, we select the combination, minimizing MSE. This result always indicates the best parameter’s combination to be used for image smoothing as the minimization error between the noise-free image and filtered image.

Consequently, we analyze the variance of individual parameters from Table 2 for individual datasets including 80 images of the fat saturation (FS) technique, 70 MR images of proton density-weighted imaging (PDw), and 40 coronal fat-saturated proton density-weighted images (FS-PDw). This analysis is provided for 9 different noise levels of Rician, Salt and Pepper, and Speckle noise. Table 3 reports the averaged values for individual tests. Based on the results, the lowest variance is mostly achieved for Rician noise, which is the most typical for MR images. This fact leads to the conclusion that the optimal filter’s settings appear to be mostly stable when Rician noise is present. On the other hand, typically higher modification of selected parameters is reported in the case of presence of Speckle noise.

### 4.4. Quantification Parameters for NLM Filter Evaluation

For each test, we evaluated a respective evaluation parameter based on the comparison between the native (noise-free) image and the result of the filter. We do not aim for providing such a comparison for single noise intensity, but the main task of this quantitative evaluation is to provide the analysis of dynamical behavior (features) of the proposed filter under various image conditions, meaning dynamic influence of the noise intensity. We consider the objectivization parameters: SNR, PSNR, Q-index and SSIM.

Signal to noise ratio (SNR) is a frequently used evaluation parameter. It indicates the relation between the power of useful image information and noise. The higher values of SNR we obtain, the less noise is present in the image. In this way, it is possible to evaluate the filtration effectivity and accurateness of noise estimation. SNR is calculated in the following way:

(15) $$SNR_{dB}=10\log_{10}\frac{\sum_{n}s^{2}\left(n\right)}{\sum_{n}{\left(s\left(n\right)-\hat{s}\left(n\right)\right)}^{2}}$$

In this definition, s(n) stands for the image after filtration and $\hat{s}\left(n\right)$ is the native image.

Quality index (Q-index) evaluates several parameters. Firstly, it evaluates a degree of linear correlation between a noisy and filtered image. In the next part, a similarity of average intensity between noisy and filtered image is evaluated. The last considered attribute is a contrast similarity. Q-index is calculated in the range 〈−1;1〉. This parameter is expressed in the following way:

(16) $$Q=\frac{\sigma_{xy}}{\sigma_{x}\sigma_{y}}\frac{2\bar{x}\bar{y}}{{\left(\bar{x}\right)}^{2}+\left(\bar{y}\right)}\frac{2\sigma_{x}\sigma_{y}}{2\sigma_{x}^{2}-\sigma_{y}^{2}}$$

In this formulation $\sigma_{xy}$ is a standard deviation between a noisy and filtered image, $\sigma_{x}\sigma_{y}$ are individual standard deviations, the parameters $\bar{x}\bar{,y}$ represent averaged values of the pixels in the mask, where Q-index is computed.

Structural Similarity Index (SSIM) is a parameter, which is aimed on the measurement of distorted image quality. This parameter compares noisy and reference image (after filtration). This parameter utilizes three attributes: contrast similarity, intensity similarity, and structural similarity. Furthermore, it is designed in such a way as to consider a human’s visual system. This parameter is normalized in the range [0;1], where higher values indicate better results. The formulation of this parameter is given:

(17) $$SSIM(x,\;y)={\left[l\left(x,y\right)\right]}^{\alpha }\cdot {\left[c\left(x,y\right)\right]}^{\beta }\cdot {\left[s\left(x,y\right)\right]}^{\gamma }$$

The parameter l(x,y) compares a similarity of intensity functions, c(x,y) signals contrast, and s(x,y) measures a structural similarity of both signals. The individual components are given by the formulations:

(18) $$l\left(x,\;y\right)=\frac{2\mu_{x}\mu_{y}+C_{1}}{\mu_{x}^{2}+\mu_{y}^{2}+C_{1}}$$

(19) $$c\left(x,\;y\right)=\frac{2\sigma_{x}\sigma_{y}+C_{2}}{\sigma_{x}^{2}+\sigma_{y}^{2}+C_{2}}$$

(20) $$s\left(x,y\right)=\frac{\sigma_{xy}+C_{3}}{\sigma_{x}\sigma_{y}+C_{3}}$$

The parameters $\mu_{x},\;\mu_{y}$ represent mean values of signals *x*, *y*, $\sigma_{x},\;\sigma_{y}$ are dispersions of signals, and $\sigma_{xy}$ stands for a mutual covariation of considered signals.

### 4.5. Filter Performance and Statistical Analysis

**Figure 6:**
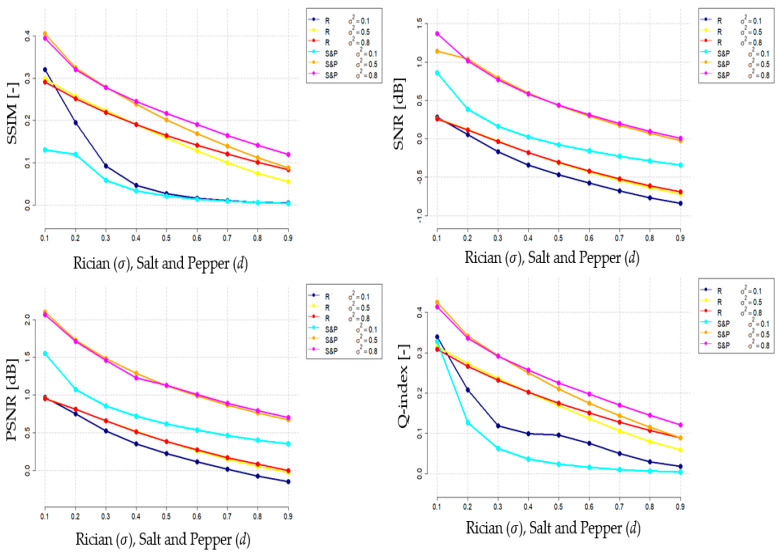
Quantitative characteristics of filter’s performance based on SSIM, SNR, PSNR and Q-index for Rician (R) and Salt and Pepper noise (SaP) with dynamic intensity for optimal filter settings, defined in Table 2 (averaged values).

In this section, we present the analysis of filter performance and a statistical analysis of the result of the proposed NLM filter against standard NLM filter, and local filtering, comprising average and median filter with various filter’s kernels. Firstly, we present the results of the filter behavior under various noise intensity levels, evaluated by the mentioned evaluation parameters, including: SSIM, SNR, PSNR, and Q-index for individual datasets. These characteristics (Figure 6) should objectively report the dynamical features of the smoothing procedure in the environment with gradually changing spatial image distribution conditions caused by the dynamic noise influence. This approach enables evaluation of the filter robustness when various noise intensity is present.

Figure 4 shows individual dynamical characteristics for considered evaluation parameters. All the characteristics are constructed for the dynamical effect of Rician noise, where we set nine various settings of σ, and Salt and Pepper noise with nine various setting of the filter’s density (d). Both noise parameters are constructed in the range: σ, d∈[0.1;0.9].

We present the characteristic for optimal filter settings, based on the optimization procedure, presented in Table 1. For contrast, we present the results of $h=\sigma^{2}=0.8$ as the best compromise from the optimization procedure against two other alternatives: $\sigma^{2}=0.1,\;0.5$ (Figure 6). These characteristics are constructed as average values of all the images from three reported datasets for 9 noise percentual levels. The dynamical trend characteristics (Figure 6) report monotonic trends for all the parameters, which should be understandable since a gradual noise influence has consecutive increasing impact on the intensity distribution in the image spatial domain. All the reported characteristics should be perceived as a similarity evaluation, meaning that the higher values we obtain, the better results we achieve (a higher level of agreement). Judging by the results, the parameter $\sigma^{2}$ has a substantial influence on the quality and robustness of the smoothing procedure. The settings $\sigma^{2}=0.8$ achieves mostly higher and thus better results when comparing with other settings. Additionally, there are noticeable differences between the considered noise generators. These are caused by the fact that both noise models have different manifestations in the image intensity distribution. When comparing the noise influence, Salt and Pepper achieves higher results when compared with Rician noise. This fact predicts a better elimination of impulse noise influence with using this smoothing procedure. Additionally, the important aspect of the evaluation is only slighter differences in Salt and Pepper noise for $\sigma^{2}=0.5,\;0.8$.

**Figure 7:**
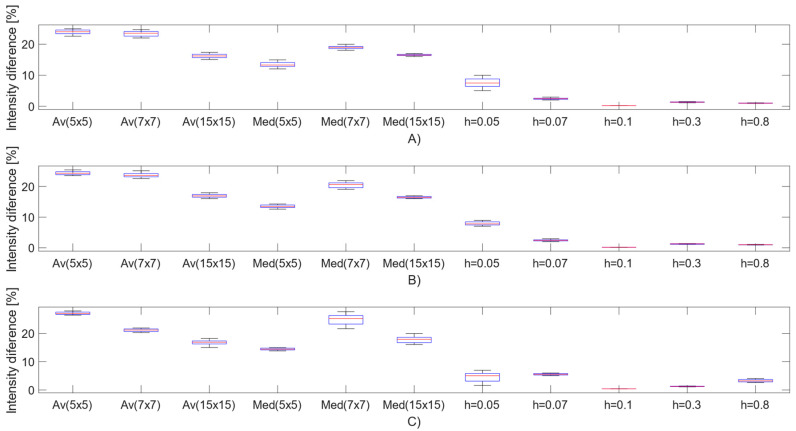
Intensity difference distribution of (**A**) Rician noise, (**B**) Salt and Pepper and (**C**) Speckle noise.

We provide a statistical analysis dealing with the intensity distribution difference between native (noise-free) and filtered images (Figure 7). This part of the statistical analysis should report the error function as intensity difference between the noise-free MR images and smoothed images via different smoothing techniques. This comparative analysis investigates the average intensity difference for all the noise levels for individual Rician, Salt and Pepper, and Speckle noise. Here, we compare local approaches, including average filter (Av), median filter (Med) and the proposed filter with four settings of the parameter (h). For the average and median filters, we compared three sizes of the filter kernel: 5 × 5, 7 × 7, and 15 × 15 kernel’s size. When comparing the results, there are noticeable intensity differences for the local mean approaches, typically in the range 10–30%, depending on the kernel’s size. In the contrast with these results, NLM filter settings for individual settings (h) do not show significant average intensity differences among each other.

The second part of the statistical analysis reports a comparison among the mentioned various settings of average and median filter in the contrast of the proposed technique with different settings (h). To objectively evaluate a statistical significance of intensity difference (ID), we calculated for each distribution two parameters of the position, including median ($\tilde{x}$) as 50% quantile and modus, reporting the most frequent value in each distribution (Mod(x)). The analysis of variance is represented by variance ($\sigma^{2}$) for each distribution of intensity difference. Table 4 provides the descriptive statistical analysis for the MR images corrupted with Rician noise.

Based on this descriptive statistical analysis, we found that the settings (h = 0.1) appear as the most effective in the context of the lowest median and modus of intensity difference and variance that show the lowest variability of intensity difference distribution. On the other hand, this statistical analysis shows significant differences of the proposed method against conventional local statistical filters such is median and average filter. In this comparison, the average filter with the kernel 5 × 5 appears to be the least effective from the view of median and modus of intensity difference. Contrarily, we found the highest variance of intensity difference in average filter 7 × 7.

We also compared the proposed optimized RNLM filter (RNLM-optim) with the RNLM filter [52]. To extend our analysis of the optimized RNLM filter, we also compared the differences between these filters on T2 mapping images of knee cartilage acquired by quantitative MRI. The data represent cases of symptomatic osteoarthritis (OA) progression. For this comparison, we used a total of 50 images of T2 maps of cartilage images, with the spatial resolution: 384 × 384 pixels, slice thickness 0.7 mm, and acquisition time of 11 min. The data acquisition was performed on 3.0 T Siemens whole body MAGNETOM Trio 3T scanner (Siemens, Erlangen, Germany), with the use of standard extremity coil.

Based on this quantitative comparison, different effectivity could be seen in the application of both filters. For the objective comparison, we selected two evaluation parameters: SSIM and correlation index (Corr). We report the objective evaluation of this parameters (Table 5) as averaged values for nine noise intensity levels as we report in Figure 4. We obtained a percentage difference between RNLM-optim and RNLM for each type of noise. Based on the averaged results for all the analyses, we found the highest differences in effectivity for Salt and Pepper noise. Moreover, the proposed optimized filter achieved the most significant results. Contrarily, in the case of Speckle noise, the differences were significantly lower.

The results between the RNLM filter and the optimized variant show notable differences in the comparison of their effectivity. In all the results, the optimized filter achieved better results, measured in difference of SSIM and correlation index. Judging by the results, for individual noise generators, the highest differences are achieved in the case of Salt and Pepper noise with impulse character. On the other hand, in the case of Speckle noise the differences were the lowest. Comparing routine anatomical imaging and T2 maps we noted slight differences in the filter effectivity. It is notable that in the case of T2 maps, differences of selected objective parameters were lower in the contrast of other MR sequences, which are considered in this study.

### 4.6. Impact on Segmentation Performance

**Figure 8:**
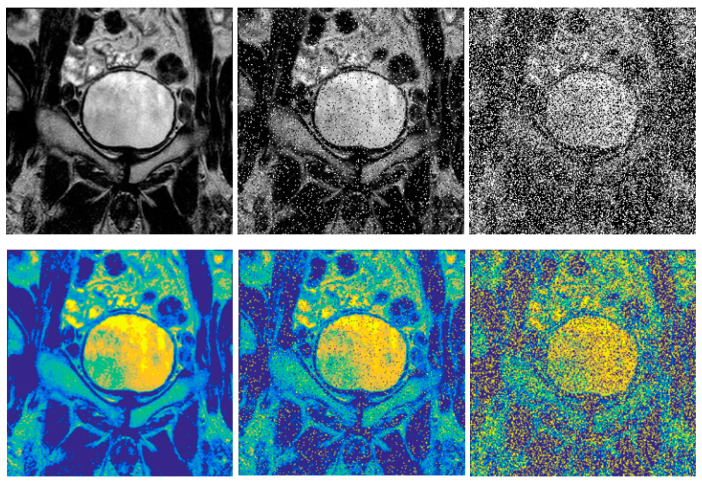
Extract of MR images: native MR image (**up left**), Salt and Pepper: d = 0.1 (**up middle**), and d = 0.5 (**up right**). Regional segmentation based on Fuzzy thresholding with 8 classes: segmentation of native image (**down left**), Salt and Pepper: d = 0.1 (**down middle**), and d = 0.5 (**down right**).

The last part of the proposed filter evaluation deals with the segmentation performance. We analyzed the effect of the smoothing procedure on the regional segmentation performance (Figure 8). Regional segmentation enables the spatial image area decomposition into a certain number of regions, which should correspond with identified objects in the image. The effectivity of this procedure is dependent on the spatial intensity distribution of individual objects of interest. When the spatial image distribution is affected with the image noise, the segmentation performance is supposed to worsen. This leads to the improper identification of the objects of interest, thus a worse quality of extracted features reporting the object’s manifestation. For the segmentation experiments, we use the concept of Fuzzy soft thresholding [46].

**Figure 9:**
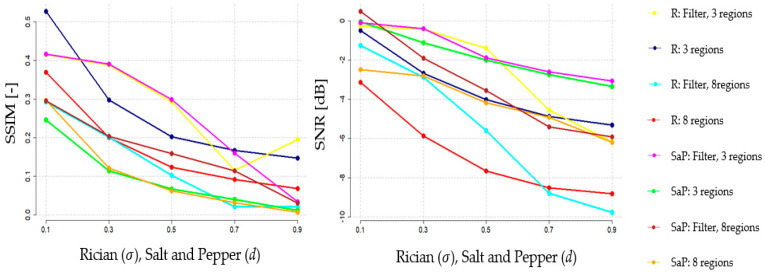
Segmentation based on Fuzzy thresholding performance (3 and 8 regions) with dynamical influence of noise Rician and Salt and Pepper based on SSIM and SNR analysis.

Here, we suppose that the increasing noise intensity will have a gradually stronger impact on the segmentation performance. We provide the analysis of the impact of various noise generators on the segmentation performance between using/not using the proposed filter for different settings of regional segmentation. Since we suppose that the number of regions should have the influence on the segmentation performance when the image noise is presented, we compare two numbers of regions, three and eight, for the filter evaluation to report how the number of regions influences the total segmentation performance between using/not using the proposed filter. We also use the evaluation parameters of SSIM and SNR for quantitative evaluation of the dynamical features of the segmentation performance under increasing noise intensity (Figure 9).

Based on the comparative analysis of the segmentation performance, it is noticeable that differences in effectivity appear. Predominantly, there are noticeable differences between the situations when the proposed filter is/is not applied. Predominantly, after applying the smoothing procedure, the segmentation performance is better, which is indicated by higher values of SSIM and SNR. We also studied the influence of the number of segmentation classes (regions) on the segmentation performance. Here, is noticeable that a lower number of classes (we use three) achieve better segmentation performance than eight classes. Regarding the dynamical trend of the segmentation performance, we can see the increasing tendency. This fact is understandable; when the noise with increasing intensity is applied, then the similarity between the native segmentation and actual noisy image segmentation is lower due to a higher modification of the spatial intensity distribution.

To contrast these quantitative results with those of the median filter, we present Table 6, a comparison between the optimized RNLM filter and the median filter with various kernels: 5 × 5, 7 × 7, and 15 × 15. All the comparisons present percentage differences between respective settings of median filter and the optimized RNLM algorithm. This comparison should report the differences between the different smoothing approaches with the influence on the regional segmentation performance. We present this comparison for Rician and Salt and Pepper noise for nine levels of the noise intensity.

Based on the results (Table 6) of the differences of SSIM between various kernel settings of the median filter and the optimized RNLM filter, notable differences are present. Firstly, all the differences report that the optimized RNLM filter contributes to better segmentation performance when compared with any median filter’s settings. When comparing the number of the segmentation classes, predominantly, a higher number of the classes (eight regions) report a higher difference in segmentation performance. This fact reports that the median filter appears to be less robust than higher numbers of the segmentation regions. The second important fact is the comparison between impulse and Rician noise from the view of the segmentation performance. In the case of impulse noise, we report a higher difference between median and optimized RNLM filter than in the case of Rician noise. That means that the impulse noise is more effectively eliminated with the effect of better segmentation performance.

## 5. Conclusions

Image smoothing is one of the essential procedures in the MR image preprocessing. This operation enables an enhancement of spatial image area by suppressing noise and artefacts, which cause image deterioration. These additive image signals lead to improper tissues identification and consequent features extraction, which is essential for proper medical diagnosis. In this context, image smoothing allows for homogenization of the intensity distribution. Conventional approaches, which are based on the local means principle, utilize a searching local window, where statistical features are computed such as an average or median filter. These methods are capable of smoothing image areas; however, on the other hand, they cause attenuation of image edges, which are crucial for tissue interpretation. In this paper, we analyzed the performance of a powerful approach, which is based on a non-local means algorithm, taking advantage of pixel intensities and patch similarity information in the contrast of these standard methods. This filter is completed with the optimization procedure, which is aimed to produce optimal filter settings. A highly important feature of each smoothing method is its robustness under a dynamically changing environment, where we suppose that the image intensity distribution is significantly modified by additive noise with dynamic intensity.

One of the main contributions of this study is studying the dynamical features of the NLM method under dynamical noise influence. To provide a robust analysis, we employed three different noise generators (Rician, Salt and Pepper and Speckle), which are determined by its parameters, controlling noise intensity. In this way, we simulate the dynamic effect of each type of noise to gradually deteriorate the MR image area. We perform the testing on the real retrospective MR image data, including the images of articular cartilage and elbow muscles images. For the objectivization of the smoothing performance, we used four qualitative parameters of similarity: SSIM, SNR, PSNR, and Q-index to evaluate the dynamical influence of each noise. Based on the testing, we evaluated the trend of all the parameters, which have decreasing tendency under gradual noise influence. This is predictable, since higher noise intensity causes a deeper modification of intensity distribution and, thus, the smoothing procedure is less effective when noise is increasing. As the next part of the testing, we were focused on the statistical evaluation of average intensity differences between native and smoothed images for all the levels of the noise. This statistical comparison mainly shows significant differences among various settings of local means approaches and the NLM concept, which achieves comparatively smaller differences than the average and median filters, which also predetermines its higher effectivity.

The last part of the analysis deals with the segmentation performance of a multiregional segmentation in the form of Fuzzy soft thresholding. Here, we studied the dynamical features of the segmentation performance when the NLM smoothing procedure is/ is not employed for Rician and Salt and Pepper noise. In nearly all the comparisons, we found that the filter presence has the impact on SSIM and SNR parameters to improve the smoothing accuracy. In this segmentation analysis, we were also focused on the segmentation settings, which may have the influence of the segmentation performance, when additive noise is present. Here, we compared two settings of the multiregional segmentation to evaluate the differences between these settings. We reported that a lower number of segmentation regions (we used three regions) indicates objectively better segmentation performance, when compared with eight regions.

The main aim of this paper was to point out on the performance of improved non-local means filter in various noise influence. This analysis has a strong potential to evaluate the dynamical features of the smoothing procedure. Since, in the MR imaging, the regional segmentation plays a crucial role with the aim to extract and identify tissues of interest, combination with an NLM filter appears to be a suitable alternative. Segmentation is typically aimed at the extraction of clinically important features, enabling a quantification of the objects of interest. In this way, the future trend in the application of NLM filters should be their influence on performance of extracting features under various image conditions. Such analysis should investigate the preciseness and reproducibility of clinically important features and their inclination to individual image noise and its intensity.

## Figures and Tables

**Table 1:** Acquisition parameters for analyzed datasets of articular cartilage and elbow muscle.

	Fat Saturation (Cartilage)	Proton Density-Weighted Imaging (Cartilage)	Fat-Saturated Proton Density-Weighted Images (Elbow Muscle)
FOV (mm)	160 × 160 × 80	160 × 160 × 80	140 × 140 × 70
Matrix size	288 × 245	288 × 245	288 × 245
Acquisition time	2:55	4:22	5:54
Slice thickness (mm)	1.5	1.5	1.5
Interslice gap (mm)	0.15	0.15	0.21
Scan mode	2D	2D	2D
Findings	Early cartilage osteoarthritis	Cartilage lesions	Healthy elbow muscle

**Table 2:** Optimal values of the filter parameters based on the testing for 80 MR images from various datasets.

Filter Parameter	n = 100	n = 1000	Average Values
*H* = $\sigma^{2}$	0.8	0.8	**0.8**
$deg^{k}$	6	7.31	**6.65**
$rad_{p}$	3	4	**3**
ω	3	5	**4**

**Table 3:** Analysis of variance of optimized filter’s parameter for individual datasets and additive noise.

Variance of Filter Parameter	Rician Noise (FS|PDW|FSPDW)			SaP Noise (FS|PDW|FSPDW)			Speckle Noise (FS|PDW|FSPDW)		
***h*** = $\sigma^{2}$	**0.05**	**0.21**	**0.08**	**0.09**	**0.32**	0.09	0.11	0.39	0.44
$deg^{k}$	0.21	0.73	0.45	0.38	0.42	0.88	0.42	0.42	0.65
$rad_{p}$	0.004	0.005	0.003	0.005	0.009	0.002	0.12	0.22	0.19
ω	0.48	0.51	0.49	0.53	0.68	0.77	0.87	0.92	0.91

**Table 4:** Descriptive statistical analysis of various average and median filter’s settings in contrast to selected variants of optimized RNLM filter for MR images corrupted with Rician noise.

Filter Settings	$\tilde{ID}\;\left[\%\right]\;$	Mod(ID) [%]	$\sigma^{2}\left(ID\right)\left[-\right]\;$
Av (5 × 5)	23.78	26.51	0.42
Av (7 × 7)	21.27	20.32	0.52
Av (15 × 15)	16.35	15.41	0.46
Med (5 × 5)	11.34	10.11	0.48
Med (7 × 7)	9.45	9.0041	0.083
Med (15 × 15)	8.31	8.011	0.021
h = 0.05	3.61	2.55	0.38
h = 0.07	1.21	1.0084	0.018
h = 0.1	0.092	0.091	1.94 × 10^6^
h = 0.3	0.65	0.55	0.0036
h = 0.8	0.45	0.45	9.46 × 10^4^

**Table 5:** A comparison for RNLM filter and proposed optimized variant based on SSIM and correlation difference for routine anatomical imaging and quantitative Magnetic Resonance Imaging (T2 maps) of cartilage.

Evaluation Parameter	Rician Noise (RNLM-Optim-RNLM)		Salt and Pepper (RNLM-Optim-RNLM)		Speckle Noise (RNLM-Optim-RNLM)	
Routine Anatomical Imaging|Quantitative Magnetic Resonance Imaging (T2 Maps)						
**Diff(SSIM)**	**12%**	8%	24%	20%	6%	5%
Diff(Corr)	15%	10%	23%	21%	8%	12%

**Table 6:** A comparison of the regional segmentation performance for two different settings of regions (segmentation classes) in application of median and optimized RNLM filter under influence of Rician and Salt and Pepper noise.

	Rician Noise (3 Classes|8 Classes)		Salt and Pepper Noise (3 Classes|8 Classes)	
Diff(SSIM(Med 5 × 5))	19.24%	14.61%	26.12%	19.55%
Diff(SSIM(Med 7 × 7))	17.87%	12.56%	21.44%	19.77%
Diff(SSIM(Med 7 × 7))	9.56%	6.15%	14.47%	12.22%
Diff(Cor(Med 7 × 7))	18.56%	17.44%	19.15%	18.86%
Diff(Cor(Med 7 × 7))	14.32%	14.11%	16.45%	15.78%
Diff(Cor(Med 7 × 7))	10.15%	9.51%	11.56%	11.12%

## Data Availability

Data are used from publicly open clinical database *The Osteoarthritis Initiative*.
